# Supplementary material for: A Likelihood Approach for Real-Time Calibration of Stochastic Compartmental Epidemic Models
Source: PLoS Comput Biol. 2017 Jan 17;13(1):e1005257. doi: 10.1371/journal.pcbi.1005257 (PMC5240920; doi:10.1371/journal.pcbi.1005257)
Supplement: S1 File — (TAR.GZ) [file pcbi.1005257.s014.tar.gz › HSPH_Online-SI-Revision/IPoi09/n1-mild/n1-mild_BENCH2_table-4.pdf]

1	newly 3 weeks spec	24.	44.8571	{9., 75.}	106.696	0.363633	newly 3 weeks cumul	78.	128.125	{27., 216.}	89.4002	0.324774	newly 5 weeks spec	32.	44.9821	{12., 69.}	66.0156	0.285952	newly 5 weeks cumul	149.	219.857	{53., 334.}	71.6922	0.286004	newly 59 weeks cumul	331.	575.179	{263., 754.}	84.6029	0.293891
2	newly 3 weeks spec	34.	15.6727	{3., 31.}	55.508	0.523474	newly 3 weeks cumul	90.	66.0909	{18., 125.}	43.6566	0.283219	newly 5 weeks spec	17.	8.92727	{1., 18.}	57.754	0.602896	newly 5 weeks cumul	145.	86.3091	{21., 160.}	47.6991	0.350415	newly 55 weeks cumul	202.	108.291	{30., 213.}	53.1593	0.408786
3	newly 3 weeks spec	20.	7.44828	{0., 18.}	85.8621	1.32924	newly 3 weeks cumul	57.	54.7586	{0., 127.}	83.0611	0.889776	newly 5 weeks spec	25.	1.27586	{0., 6.}	94.8966	2.15213	newly 5 weeks cumul	111.	58.3793	{0., 147.}	66.9773	1.01655	newly 64 weeks cumul	238.	58.8966	{0., 147.}	77.7166	1.29776
4	newly 3 weeks spec	15.	38.4737	{12., 67.}	172.982	0.51279	newly 3 weeks cumul	42.	113.895	{44., 178.}	184.712	0.550151	newly 5 weeks spec	9.	35.5526	{5., 56.}	313.158	0.713232	newly 5 weeks cumul	71.	183.711	{55., 274.}	174.388	0.560204	newly 58 weeks cumul	398.	396.105	{57., 598.}	35.242	0.350525
5	newly 3 weeks spec	50.	19.5833	{0., 44.}	64.6111	0.927485	newly 3 weeks cumul	122.	90.7778	{11., 189.}	53.051	0.481185	newly 5 weeks spec	37.	7.83333	{0., 26.}	80.3303	1.44827	newly 5 weeks cumul	231.	111.556	{11., 228.}	56.9024	0.625864	newly 60 weeks cumul	369.	121.944	{11., 259.}	67.4947	0.786849
6	newly 3 weeks spec	26.	11.6304	{0., 26.}	67.9766	0.839391	newly 3 weeks cumul	47.	28.8043	{1., 69.}	68.1314	0.689215	newly 5 weeks spec	37.	15.8913	{0., 42.}	72.7967	1.02218	newly 5 weeks cumul	121.	58.8261	{1., 135.}	67.4811	0.813265	newly 64 weeks cumul	400.	440.63	{1., 786.}	80.2011	0.885173
7	newly 3 weeks spec	25.	12.1071	{0., 29.}	65.8571	0.933892	newly 3 weeks cumul	44.	65.9286	{3., 153.}	100.974	0.464433	newly 5 weeks spec	48.	8.96429	{0., 18.}	93.9732	1.91134	newly 5 weeks cumul	142.	83.2857	{3., 192.}	60.9155	0.609849	newly 63 weeks cumul	381.	107.071	{3., 281.}	75.2718	0.946168
8	newly 3 weeks spec	13.	24.1707	{6., 41.}	116.323	0.437447	newly 3 weeks cumul	44.	66.3171	{14., 109.}	87.7494	0.363872	newly 5 weeks spec	14.	23.3415	{7., 40.}	93.9024	0.39117	newly 5 weeks cumul	78.	112.073	{19., 181.}	78.3302	0.348609	newly 55 weeks cumul	449.	453.463	{317., 611.}	24.7107	0.164856
9	newly 3 weeks spec	32.	24.225	{7., 47.}	44.1406	0.2732	newly 3 weeks cumul	72.	88.	{44., 154.}	46.5972	0.175159	newly 5 weeks spec	42.	17.	{3., 36.}	61.3095	0.552199	newly 5 weeks cumul	158.	124.775	{55., 210.}	38.2437	0.217432	newly 54 weeks cumul	322.	201.7	{69., 387.}	51.7391	0.353973
10	newly 3 weeks spec	37.	41.3871	{18., 67.}	42.1099	0.182907	newly 3 weeks cumul	113.	141.161	{68., 205.}	44.5618	0.172208	newly 5 weeks spec	27.	29.4677	{9., 52.}	47.491	0.248184	newly 5 weeks cumul	184.	206.581	{90., 316.}	39.9018	0.172679	newly 55 weeks cumul	266.	332.177	{124., 503.}	54.5901	0.218432
11	newly 3 weeks spec	20.	19.4839	{6., 31.}	40.9677	0.273165	newly 3 weeks cumul	44.	53.4194	{13., 96.}	59.6774	0.28841	newly 5 weeks spec	18.	20.4839	{5., 33.}	50.	0.295585	newly 5 weeks cumul	86.	93.4839	{27., 148.}	47.862	0.275364	newly 54 weeks cumul	455.	446.161	{224., 618.}	26.0475	0.222905
12	newly 3 weeks spec	14.	11.7647	{0., 30.}	68.9076	0.568308	newly 3 weeks cumul	27.	34.9412	{2., 87.}	86.0566	0.456886	newly 5 weeks spec	18.	12.0588	{0., 29.}	61.1111	0.707676	newly 5 weeks cumul	68.	57.8235	{2., 153.}	63.2353	0.526313	newly 55 weeks cumul	321.	103.235	{2., 228.}	68.6458	0.885999
13	newly 3 weeks spec	16.	26.5818	{7., 60.}	93.6364	0.27069	newly 3 weeks cumul	60.	103.727	{42., 212.}	89.1818	0.246571	newly 5 weeks spec	15.	17.0727	{4., 40.}	81.697	0.40815	newly 5 weeks cumul	111.	141.673	{54., 300.}	64.783	0.236743	newly 56 weeks cumul	193.	182.782	{60., 446.}	58.6528	0.293128
14	newly 3 weeks spec	29.	62.7755	{14., 93.}	131.949	0.421164	newly 3 weeks cumul	93.	201.918	{107., 273.}	128.22	0.414768	newly 5 weeks spec	30.	46.5714	{9., 84.}	81.0884	0.360554	newly 5 weeks cumul	159.	302.694	{125., 416.}	102.849	0.370117	newly 60 weeks cumul	260.	469.612	{221., 650.}	91.5777	0.341345
15	newly 3 weeks spec	25.	25.8286	{6., 61.}	67.0857	0.348851	newly 3 weeks cumul	71.	94.6286	{39., 169.}	61.3682	0.229491	newly 5 weeks spec	20.	14.0286	{0., 40.}	76.1429	0.797151	newly 5 weeks cumul	129.	128.886	{47., 236.}	50.8527	0.25846	newly 60 weeks cumul	389.	191.	{47., 449.}	58.318	0.50254
16	newly 3 weeks spec	23.	55.5517	{29., 81.}	146.627	0.375265	newly 3 weeks cumul	52.	159.534	{69., 220.}	212.566	0.476671	newly 5 weeks spec	7.	50.8793	{26., 70.}	626.847	0.835521	newly 5 weeks cumul	83.	264.103	{161., 347.}	221.396	0.490624	newly 58 weeks cumul	349.	581.621	{421., 718.}	69.4892	0.223555
17	newly 3 weeks spec	17.	43.52	{7., 66.}	177.176	0.572532	newly 3 weeks cumul	43.	123.34	{32., 187.}	203.395	0.60221	newly 5 weeks spec	15.	40.88	{0., 68.}	198.933	0.641578	newly 5 weeks cumul	78.	208.08	{36., 309.}	184.718	0.601921	newly 61 weeks cumul	332.	475.94	{36., 717.}	73.006	0.480767
18	newly 3 weeks spec	18.	32.7755	{13., 52.}	96.3719	0.314545	newly 3 weeks cumul	52.	93.4898	{38., 143.}	94.0738	0.305608	newly 5 weeks spec	18.	32.5306	{12., 56.}	96.1451	0.338141	newly 5 weeks cumul	99.	160.061	{71., 244.}	76.6028	0.276364	newly 54 weeks cumul	320.	459.714	{241., 646.}	54.5153	0.213256
19	newly 3 weeks spec	40.	78.3284	{34., 109.}	104.403	0.305199	newly 3 weeks cumul	125.	210.731	{76., 313.}	83.8448	0.269087	newly 5 weeks spec	35.	79.3881	{54., 101.}	126.823	0.344394	newly 5 weeks cumul	206.	371.731	{200., 466.}	85.0022	0.260368	newly 61 weeks cumul	389.	757.478	{637., 871.}	94.7243	0.286217
20	newly 3 weeks spec	37.	48.7368	{14., 76.}	55.2394	0.239241	newly 3 weeks cumul	71.	134.965	{34., 217.}	108.821	0.340791	newly 5 weeks spec	33.	48.1228	{22., 68.}	61.0314	0.248038	newly 5 weeks cumul	150.	234.018	{84., 332.}	73.1579	0.270238	newly 58 weeks cumul	270.	621.07	{468., 763.}	133.821	0.375706
21	newly 3 weeks spec	20.	4.5	{0., 15.}	87.5	1.87093	newly 3 weeks cumul	49.	12.1	{0., 51.}	83.0612	2.00662	newly 5 weeks spec	20.	0.8	{0., 1.}	96.	2.01652	newly 5 weeks cumul	108.	14.8	{0., 67.}	86.2963	2.30196	newly 58 weeks cumul	233.	15.7	{0., 76.}	93.2618	2.63042
22	newly 3 weeks spec	7.	40.6275	{14., 63.}	486.555	0.748278	newly 3 weeks cumul	28.	113.647	{37., 184.}	311.905	0.571601	newly 5 weeks spec	13.	43.8627	{23., 61.}	242.232	0.529183	newly 5 weeks cumul	64.	199.863	{81., 283.}	218.413	0.480487	newly 60 weeks cumul	439.	604.078	{418., 748.}	40.1134	0.14165
23	newly 3 weeks spec	19.	6.95122	{0., 20.}	70.8601	0.90073	newly 3 weeks cumul	44.	27.0976	{2., 66.}	66.4634	0.680948	newly 5 weeks spec	23.	4.68293	{0., 17.}	83.4571	1.28866	newly 5 weeks cumul	99.	36.5854	{2., 96.}	71.4708	0.864642	newly 55 weeks cumul	317.	53.6341	{3., 145.}	83.2346	1.23147
24	newly 3 weeks spec	25.	39.3269	{13., 60.}	75.6154	0.273584	newly 3 weeks cumul	65.	114.077	{39., 172.}	93.7278	0.311782	newly 5 weeks spec	28.	39.9231	{16., 60.}	60.3022	0.236849	newly 5 weeks cumul	129.	194.885	{86., 278.}	67.4717	0.253843	newly 57 weeks cumul	399.	512.827	{300., 722.}	42.9391	0.179158
25	newly 3 weeks spec	15.	22.5405	{4., 45.}	87.027	0.349838	newly 3 weeks cumul	35.	73.8919	{20., 138.}	127.336	0.348444	newly 5 weeks spec	9.	18.8378	{2., 34.}	137.538	0.439889	newly 5 weeks cumul	63.	115.595	{38., 228.}	103.99	0.31582	newly 54 weeks cumul	293.	203.216	{53., 376.}	50.3828	0.370143
26	newly 3 weeks spec	28.	49.5319	{23., 72.}	93.1611	0.365773	newly 3 weeks cumul	64.	149.043	{63., 213.}	148.438	0.450348	newly 5 weeks spec	24.	43.5106	{9., 67.}	100.798	0.315159	newly 5 weeks cumul	119.	239.085	{156., 344.}	115.323	0.371524	newly 57 weeks cumul	217.	521.596	{266., 706.}	140.955	0.3589
27	newly 3 weeks spec	24.	33.7358	{13., 51.}	61.7925	0.238229	newly 3 weeks cumul	69.	95.3585	{33., 155.}	61.3344	0.23388	newly 5 weeks spec	20.	31.7547	{18., 45.}	67.2642	0.229736	newly 5 weeks cumul	118.	159.642	{73., 237.}	53.4538	0.205122	newly 54 weeks cumul	267.	570.321	{427., 705.}	113.603	0.322039
28	newly 3 weeks spec	11.	15.3571	{2., 38.}	101.515	0.465396	newly 3 weeks cumul	24.	40.6905	{4., 98.}	122.123	0.497552	newly 5 weeks spec	16.	16.881	{1., 33.}	67.7083	0.474556	newly 5 weeks cumul	66.	73.119	{8., 155.}	76.7316	0.488433	newly 60 weeks cumul	343.	495.19	{174., 710.}	68.8602	0.43397
29	newly 3 weeks spec	36.	41.06	{17., 61.}	39.9444	0.177281	newly 3 weeks cumul	105.	132.1	{52., 190.}	48.3238	0.203179	newly 5 weeks spec	22.	37.98	{12., 57.}	87.9091	0.280599	newly 5 weeks cumul	167.	212.76	{99., 296.}	45.1737	0.182857	newly 57 weeks cumul	288.	463.72	{194., 679.}	73.1667	0.239882
30	newly 3 weeks spec	28.	71.451	{31., 100.}	166.387	0.46507	newly 3 weeks cumul	83.	207.725	{72., 294.}	163.407	0.444424	newly 5 weeks spec	27.	58.4706	{32., 85.}	123.965	0.338733	newly 5 weeks cumul	148.	332.824	{154., 437.}	132.538	0.371212	newly 61 weeks cumul	335.	610.961	{390., 809.}	83.7109	0.251834
31	newly 3 weeks spec	56.	58.8103	{24., 89.}	34.1441	0.15958	newly 3 weeks cumul	151.	188.931	{93., 284.}	41.288	0.165129	newly 5 weeks spec	34.	47.0345	{16., 85.}	66.2272	0.254612	newly 5 weeks cumul	244.	289.776	{149., 394.}	37.5565	0.154552	newly 59 weeks cumul	353.	499.017	{229., 762.}	55.529	0.194383
32	newly 3 weeks spec	11.	29.8261	{8., 49.}	188.142	0.531891	newly 3 weeks cumul	27.	83.6304	{11., 139.}	230.515	0.619638	newly 5 weeks spec	24.	31.3696	{9., 54.}	58.9674	0.380525	newly 5 weeks cumul	76.	144.37	{26., 222.}	114.731	0.500123	newly 57 weeks cumul	391.	472.457	{155., 709.}	43.4727	0.394708
33	newly 3 weeks spec	37.	47.4468	{23., 72.}	44.1058	0.174253	newly 3 weeks cumul	84.	130.702	{67., 193.}	67.0973	0.225948	newly 5 weeks spec	32.	48.0213	{22., 73.}	61.5027	0.205358	newly 5 weeks cumul	159.	227.957	{133., 341.}	52.7365	0.184918	newly 58 weeks cumul	332.	580.106	{410., 749.}	77.6403	0.242655
34	newly 3 weeks spec	13.	6.71875	{0., 17.}	76.2019	1.03019	newly 3 weeks cumul	35.	29.4375	{0., 78.}	78.0357	0.891264	newly 5 weeks spec	9.	5.5	{0., 18.}	88.1944	1.15007	newly 5 weeks cumul	63.	41.6563	{0., 115.}	74.0575	1.00926	newly 57 weeks cumul	230.	62.5625	{0., 137.}	79.1033	1.40075
35	newly 3 weeks spec	24.	47.75	{13., 72.}	120.486	0.408402	newly 3 weeks cumul	77.	128.633	{23., 203.}	91.6883	0.35627	newly 5 weeks spec	30.	52.4833	{20., 72.}	89.5	0.320748	newly 5 weeks cumul	154.	234.15	{69., 341.}	73.7121	0.312821	newly 59 weeks cumul	405.	658.633	{498., 783.}	69.2428	0.29553
36	newly 3 weeks spec	51.	65.15	{31., 94.}	47.3529	0.201666	newly 3 weeks cumul	141.	167.017	{57., 251.}	47.6005	0.216598	newly 5 weeks spec	46.	72.3833	{46., 93.}	62.0652	0.207065	newly 5 weeks cumul	242.	308.65	{147., 414.}	42.7893	0.17408	newly 61 weeks cumul	339.	738.917	{578., 848.}	117.97	0.334581
37	newly 3 weeks spec	18.	12.8571	{0., 26.}	60.7143	0.713624	newly 3 weeks cumul	52.	46.7857	{2., 92.}	59.6154	0.497858	newly 5 weeks spec	33.	9.21429	{0., 21.}	82.2511	1.17829	newly 5 weeks cumul	122.	67.6071	{2., 126.}	55.0644	0.625779	newly 57 weeks cumul	268.	107.929	{2., 282.}	70.5757	0.85258
38	newly 3 weeks spec	43.	75.3594	{47., 101.}	81.5044	0.252409	newly 3 weeks cumul	95.	234.891	{142., 314.}	148.635	0.378748	newly 5 weeks spec	60.	63.625	{30., 92.}	34.4792	0.163509	newly 5 weeks cumul	220.	369.703	{256., 469.}	70.7884	0.225206	newly 61 weeks cumul	371.	603.422	{379., 847.}	68.1393	0.220337
39	newly 3 weeks spec	35.	27.5882	{5., 66.}	57.6471	0.413126	newly 3 weeks cumul	95.	108.412	{42., 173.}	57.709	0.252608	newly 5 weeks spec	16.	18.7647	{1., 48.}	84.5588	0.515006	newly 5 weeks cumul	151.	150.029	{47., 292.}	52.3763	0.267026	newly 59 weeks cumul	278.	204.088	{49., 446.}	52.6132	0.355702
40	newly 3 weeks spec	40.	15.56	{0., 53.}	73.7	1.02753	newly 3 weeks cumul	91.	72.48	{3., 157.}	55.1648	0.524445	newly 5 weeks spec	51.	7.32	{0., 36.}	86.902	1.89232	newly 5 weeks cumul	193.	90.72	{3., 258.}	64.7668	0.726391	newly 62 weeks cumul	329.	101.24	{3., 294.}	69.7629	0.89283
41	newly 3 weeks spec	26.	70.7333	{22., 110.}	182.436	0.441011	newly 3 weeks cumul	100.	168.8	{40., 273.}	87.3333	0.295535	newly 5 weeks spec	38.	81.8167	{37., 113.}	122.851	0.349917	newly 5 weeks cumul	177.	328.35	{132., 481.}	95.904	0.299353	newly 63 weeks cumul	284.	741.683	{575., 889.}	161.673	0.408351
42	newly 3 weeks spec	58.	34.4545	{4., 70.}	50.9404	0.547348	newly 3 weeks cumul	126.	107.818	{19., 194.}	48.4367	0.386849	newly 5 weeks spec	44.	30.697	{1., 71.}	58.7466	0.639987	newly 5 weeks cumul	229.	170.121	{37., 315.}	43.8666	0.417148	newly 62 weeks cumul	291.	320.545	{45., 629.}	71.6547	0.49106
43	newly 3 weeks spec	61.	101.895	{34., 163.}	84.9871	0.280522	newly 3 weeks cumul	155.	330.544	{206., 420.}	115.382	0.319428	newly 5 weeks spec	49.	53.6316	{6., 107.}	65.8074	0.394471	newly 5 weeks cumul	274.	462.772	{231., 614.}	76.4631	0.243512	newly 64 weeks cumul	392.	547.632	{257., 799.}	54.8917	0.200086
44	newly 3 weeks spec	34.	50.9231	{29., 73.}	61.1991	0.242598	newly 3 weeks cumul	64.	157.077	{83., 212.}	152.043	0.434079	newly 5 weeks spec	35.	40.6154	{17., 60.}	39.6703	0.205611	newly 5 weeks cumul	143.	244.173	{136., 335.}	77.2324	0.290741	newly 56 weeks cumul	211.	455.788	{257., 662.}	120.552	0.383025
45	newly 3 weeks spec	33.	34.3725	{18., 50.}	29.8277	0.138937	newly 3 weeks cumul	81.	115.451	{73., 155.}	50.8594	0.176846	newly 5 weeks spec	24.	28.3725	{10., 50.}	49.9183	0.216928	newly 5 weeks cumul	141.	176.275	{106., 240.}	39.3687	0.150851	newly 54 weeks cumul	301.	302.667	{131., 521.}	39.2222	0.18261
46	newly 3 weeks spec	19.	49.2	{6., 78.}	184.372	0.547677	newly 3 weeks cumul	57.	127.538	{10., 211.}	152.254	0.518925	newly 5 weeks spec	23.	54.9077	{9., 79.}	161.472	0.503475	newly 5 weeks cumul	104.	233.246	{28., 355.}	149.364	0.511387	newly 61 weeks cumul	451.	671.569	{542., 808.}	58.4513	0.315642
47	newly 3 weeks spec	5.	21.2857	{4., 47.}	337.143	0.552197	newly 3 weeks cumul	19.	57.9143	{10., 126.}	222.857	0.459948	newly 5 weeks spec	12.	27.6286	{5., 48.}	156.429	0.483281	newly 5 weeks cumul	47.	109.571	{16., 212.}	155.988	0.419017	newly 59 weeks cumul	335.	580.914	{396., 717.}	79.0874	0.299977
48	newly 3 weeks spec	15.	25.8205	{9., 40.}	88.547	0.266536	newly 3 weeks cumul	52.	89.4872	{39., 153.}	81.2623	0.242659	newly 5 weeks spec	38.	18.1795	{3., 38.}	54.3185	0.495712	newly 5 weeks cumul	137.	129.744	{50., 210.}	37.9001	0.189922	newly 54 weeks cumul	271.	227.744	{69., 436.}	45.4631	0.268141
49	newly 3 weeks spec	12.	23.3824	{5., 43.}	119.363	0.422265	newly 3 weeks cumul	36.	69.7353	{15., 132.}	118.873	0.399041	newly 5 weeks spec	11.	23.9118	{8., 39.}	137.166	0.46282	newly 5 weeks cumul	67.	118.5	{35., 212.}	101.273	0.390667	newly 58 weeks cumul	292.	431.971	{219., 662.}	65.5822	0.333374
50	newly 3 weeks spec	24.	50.7255	{37., 70.}	119.526	0.358434	newly 3 weeks cumul	70.	150.157	{102., 194.}	123.473	0.363499	newly 5 weeks spec	19.	45.2549	{26., 68.}	142.312	0.39703	newly 5 weeks cumul	120.	244.529	{180., 322.}	110.866	0.337812	newly 55 weeks cumul	321.	499.647	{310., 690.}	62.0793	0.226201
